# General practitioners’ perceptions of their role and their collaboration with district nurses in wound care

**DOI:** 10.1017/S1463423618000464

**Published:** 2018-07-19

**Authors:** Anne Friman, Desiree Wiegleb Edström, Samuel Edelbring

**Affiliations:** 1 PhD candidate, Department of Neurobiology, Care Sciences and Society, Karolinska Institutet, Huddinge, Sweden; 2 Department of Learning, Informatics, Management and Ethics, Karolinska Institutet, Huddinge, Sweden; 3 School of Education, Health and Social Studies, Dalarna University, Falun, Sweden; 4 Associate Professor of Dermatology, Senior Lecturer in Medical Education and Senior Consultant responsible for the treatment section, Dermatology Unit, Department of Medicine Solna, Karolinska Institutet, Huddinge, Sweden; 5 Department of Dermatology, Karolinska University Hospital, Stockholm, Sweden; 6 Associate Senior Lecturer in Medical Education, Faculty of Medicine and Health Sciences, Linköping University, Linköping, Sweden

**Keywords:** collaboration, GP, professional role, wound care

## Abstract

**Aim:**

To explore the perceptions of general practitioners (GPs) regarding their role and their collaboration with district nurses (DNs) in the management of leg ulcers in primary healthcare.

**Background:**

Earlier research regarding the treatment of leg ulcers in a primary care context has focussed primarily on wound healing. Less is known about GPs’ understandings of their role and their collaboration with DNs in the management of leg ulcers. Since the structured care of patients with leg ulcers involving both GP and DN is currently rather uncommon in Swedish primary care, this study sets out to highlight these aspects from the GP’s perspective.

**Methods:**

Semi-structured individual interviews with 16 GPs including both private and county council run healthcare centres. Thematic analysis was used to analyse the data.

**Results:**

Four themes were identified. The first theme: ‘role as consultant and coordinator’ shows how the GPs perceived their role in wound care. In the second theme: ‘responsibility for diagnosis’ the GPs’ views on responsibility for wound diagnosis is presented. The third theme: ‘desire for continuity’ is based on the GPs’ desire for continuity concerning various aspects. In the fourth theme: ‘collaboration within the organisation’ the importance of the organisation for collaboration between GPs and DNs is presented.

**Conclusions:**

The GP’s often work on a consultation-like basis and feel that they become involved late in the patients’ wound treatment. This can have negative consequences for the medical diagnosis and, thereby, lead to a prolonged healing time for the patient. Shortcomings regarding collaboration are mainly attributed to organisational factors.

## Background

People with multiple diagnoses require collaboration and interplay between different competences in today’s healthcare (Moore *et al*., 2014; Swedish Agency for Health Technology Assessment and Assessment of Social Services [SBU], 2014). One such arena is management of patients suffering from chronic leg ulcers in which general practitioners (GPs) and district nurses (DNs) are prominent actors in primary care (Sadler *et al*., 2006; Mooij and Huisman, 2016). Chronic leg ulceration is a condition of the lower limb resulting from altered physiology of the blood vessels which can include the veins, arteries or both; most commonly the veins as a result of chronic hypertension associated with incompetent valves in the deep and perforating veins (Velnar *et al*., 2009; SBU, 2014; Franks *et al*., 2016). The wound healing is due to the underlying aetiology. The highest prevalence of leg ulceration is stated to be of venous origin (Vowden and Vowden, 2009; Gottrup *et al*., 2013; Guest *et al*., 2017).

The risk of developing leg ulcers is expected to increase with advanced age, which is important in the current demographic situation in Europe with an ageing population (Forssgren *et al*., 2008; Forsgren and Nelzén, 2012; SBU, 2014). Leg ulcers take a long time to heal, on average 12–13 months, and they often recur (Moffat *et al*., 2010; Franks *et al*., 2016). Significant amounts of time and resources are invested in the healing of leg ulcers, with developed countries spending approximately 2–5% of their total healthcare costs on the condition (Ragnarson Tennvall *et al*., 2004; Öien and Ragnarson Tennvall, 2006; Edwards *et al*., 2013; Phillips *et al*., 2015).

Leg ulcers affect the patient’s life in various ways such as through pain and sleep impairment, and they may be foul-smelling which can lead to social isolation, thus affecting the patient’s quality of life negatively (Herber *et al*., 2007; Phillips *et al*., 2017).

A number of guidelines aim to ensure optimal leg ulcer treatment (Scottish Intercollegiate Guidelines Network [SIGN], 2010; Wound, Ostomy and Continence Nurses Society [WOCN], 2013; VISS, 2016). According to these guidelines, appropriate wound assessment requires a thorough patient history and clinical examination, including Doppler assessment, to evaluate circulation and healing conditions, which together contribute to determining the aetiologic diagnosis of the wound and form the basis of a treatment plan with information about the focus of the care and treatment (Sinha and Sreedharan, 2014; Mooij and Huisman, 2016). A wound assessment, an aetiologic diagnosis and an investigation of the underlying causes of wounds should be made by a physician, while the treatment of leg ulcers is primarily performed by a DN, always in collaboration with a GP (VISS, 2016).

Several studies, both Swedish and international, have identified shortcomings in the wound care provided in primary care, in particular in relation to the assessment of wounds (McGuckin and Kerstein, 1998; Öien and Ragnarson Tennvall, 2006; Mooij and Huisman, 2016). It has been found that, in primary care, wounds are generally treated without an established aetiologic diagnosis, which is thought to be due to insufficient collaboration between GPs and DNs (Friman *et al*., 2010; Sinha and Sreedharan, 2014; Mooij and Huisman, 2016).

Various causes of insufficient collaboration have been presented in the literature. McInness *et al*. (2015) emphasise homogeneity in education, suggesting that the separate education of nurses and doctors can have a negative effect on the ability of these professions to collaborate in a team. Studies in Sweden have shown a fundamental ambivalence by GPs to share the overall responsibility for patient care with other healthcare professionals (Hansson *et al*., 2008; Hansson *et al*., 2010). Further barriers to collaboration that are emphasised include organisational factors such as lack of clarity regarding leadership, status, professional socialisation and responsibility for decisions (Baranoski, 1992; Whitehead, 2007; Milburn and Colyer, 2008; Xyrichis and Lowton, 2008). In general, there appears to be lack of definition regarding roles and sharing of responsibility, which clearly impedes collaboration in primary care (McInnes *et al*., 2015; Mooij and Huisman, 2016).

Previous studies have found that interprofessional collaboration can lead to improved wound healing (Krishnan *et al*., 2008; Chiu *et al*., 2011; Marola *et al*., 2016). This is because different professions contribute with their respective knowledge and skills in both wound assessment and treatment (Moore *et al*., 2014). Studies of collaboration in wound treatment have been conducted primarily with a focus on wound healing. Less is known about GPs understandings of their role and collaboration with DNs in wound care. Since the structured care of patients with leg ulcers involving both GPs and DNs is currently rather uncommon in Swedish primary care, this study sets out to highlight these aspects from the GP’s perspective.

## Research question

How do GPs understand their role and their collaboration with district nurses in wound care?

## Research methods

A qualitative descriptive design with individual interviews was chosen as appropriate for describing how GPs’ experience their role in wound care and their collaboration with DNs in primary healthcare centres. This design was selected because it provides the opportunity to gain rich and meaningful data on individual experiences and allows informants to freely express their views on the topic (Patton, 2002). Qualitative interviews were conducted with 16 GPs at different healthcare centres, that is, at the respective GP’s workplace. A semi-structured interview guide with question areas regarding role and collaboration in wound care was used to assist in the interviews. Question areas with possible follow-up questions were pilot tested in five interviews to check the relevance of the questions (Patton, 2002; Kvale and Brinkmann, 2009). Two registered nurses conducted these interviews. These nurses attended a specialist education for district nurses and needed practice in qualitative interview methods. Refinements to the interview guide were made after the pilot interviews, for example, the specification of the follow-up questions regarding role and collaboration. The interviews lasted ~30 min and were recorded and transcribed verbatim. All authors participated in the data collection (Table 1).

**Table 1:** Interview guide

Question areas	Main question with follow-up questions
GPs role in the treatment of chronic leg ulcers	How do you consider your role as GP in the treatment of chronic leg ulcers? Examples of follow-up questions: Can you tell me about the types of leg ulcers you have treated lately? How was it to diagnose the wound? Do you want to describe the diagnosis process? Can you describe the difficulties in the process? What was easy? How did you reach a decision on treatment? How do you manage the local treatment?
Collaboration in wound care	How do you consider the importance of working with other professions in wound treatment? Examples of follow-up questions: How do DNs and GPs work at your healthcare centre in the treatment of chronic leg ulcers? How do you perceive that collaboration? Can you describe if there are any barriers to that collaboration? How do you think the collaboration benefits patient care?

GPs=general practitioners; DNs=district nurses

## Context/participants

Primary care in Sweden includes both private and county council owned healthcare centres. The core activities of these healthcare centres include the provision of outpatient clinics for planned (time-booked) and unplanned medical and healthcare within general medicine, including rehabilitation, psychosocial activities, health promotion and disease prevention. An out of hours service is also provided (Swedish National Board of Health and Welfare [Socialstyrelsen], 2016). The average number of patients registered per GP varies from approximately 1400 to 2000 depending on where in the country the GP works (Pettersson and Engblom, 2015).

In the present study, in order to provide variation in data, informants were recruited from both county council and privately run healthcare centres located in both the urban area of Stockholm and a medium-sized town in Sweden. The informants were GPs working at primary healthcare centres, including six men and 10 women aged from 39 to 65 years (median=49). They had worked as registered doctors for between 10 and 37 years (median=17.5) and as specialists in general practice from one to 31 years (median=8). One of the informants was also specialised in geriatrics. Informants were recruited through practice managers who selected GPs with experience of treating leg ulcers, that is, they had patients registered with them who had chronic leg ulcers.

## Ethical issues

The local Ethics Committee approved the study (Registration number 2014/615-31/1). Prior to the interviews, participants were informed regarding the aim of the study and that confidentiality was guaranteed. They were assured that they could withdraw from the study at any time.

## Data analysis

Processing and analysis of the individual interviews was carried out using thematic analysis (Braun and Clarke, 2006). Each individual interview comprised a unit of analysis and was analysed separately. First, the material was read through repeatedly to become familiar with the data. Thereafter, words and sentences in the transcripts were coded in relation to the aim of the study. The codes were then interpreted and collated into potential themes. The potential themes were considered in relation to the data set and the final themes were formed in an iterative process checking themes in relation to codes and the interview data. The primary phase of the analysis was performed by the first author (A.F.), involving D.W.E. creating themes and S.E. in secondary stages critically scrutinising the interpretations.

## Results

The analysis resulted in four themes: ‘role as consultant and coordinator’, ‘responsibility for diagnosis’, ‘desire for continuity’ and ‘collaboration within the organisation’. The themes are presented and illustrated with citations from the various interviews. The numbering of the GPs in the citations indicates the order of the interviews.

### Role as consultant and coordinator

The GPs perceived their role in wound care as being secondary; they were primarily consulted when wound healing did not proceed as expected and when it was necessary to involve other healthcare professionals. Most of the informants regarded wound care, for example, the type of dressings used, as primarily the responsibility of the DNs, whose assessment and judgement they relied on, in particular regarding wound dressings. They highlighted that patients with wounds asked to see the DNs in the first instance, and many of the informants thought that there was a kind of initial sorting when the patient contacted the healthcare centre whereby these patients were directed to the DNs. The GPs perceived wound care as fairly labour intensive and time-consuming, especially with regard to the dressing of wounds. The informants believed that this could be one reason why these patients were directed to the DNs. The DN was seen as taking the main responsibility for wound care:

‘I guess it’s because we are not responsible for the management of leg ulcers, it is in some way the nurses who are. We are of course the patients’ accountable GPs but we don’t manage the wound care, as we don’t prescribe the specific dressings etc. The nurses carry out the care on their own … we are therefore a bit more peripheral in comparison to, for example, the treatment of heart failure’. (GP 12)

The GPs stated that the DNs first called them in when the healing process was not proceeding as it should. One reason for this, according to the GPs, could be that the leg ulcer was infected:

‘I am called to the clinic when they [the patients] come to see the district nurse … I’m often called in if a wound is infected’. (GP 16)

Another reason as to why the GPs would be consulted in wound care was if the patient needed further interventions in their treatment. Examples of these interventions given by the informants were hyperbaric oxygen therapy for patients with diabetic foot ulcers, peripheral vascular circulatory assessment to diagnose the wound, treatment of eczema around the wound and compression therapy. These interventions require different skills which is why the informants regarded their role in such situations as coordinating the different interventions in the patient’s treatment:

‘we had a lady here who had a venous leg ulcer … none of us thought that her leg was particularly swollen and so she came here for treatment for a long period of time and nothing happened, so in the end I sent a referral to the leg ulcer clinic … then we received a referral report stating that compression therapy usually gives good results even if the patient doesn’t seem to have much oedema so they [DNs] bandaged her leg over a period of time and it improved and it was a positive collaboration with the dermatology department who gave sound help and advice and it was a good result for the patient’. (GP 7)

The role of coordinator was also particularly apparent in the treatment of patients with diabetes, where for example, there could be a risk of osteitis requiring an X-ray assessment. In such cases, there was sometimes a need for a referral for further examinations and investigations. The informants considered that, in these cases, they had an important advisory role particularly in patients with diabetes who had developed leg ulcers, and where the DN requested an assessment of the wound:

‘to assess and diagnose what is the root cause of the leg ulcer, is it an undiagnosed diabetes … it is important to rule out diabetes with delayed wound healing’. (GP 6)

The GPs consistently saw their role in wound care as that of a consultant who seldom sees patients with wounds. One informant expressed it in this way:

‘we are consultants. We are consulted when they think it is not going as planned so that – say, I maybe see a patient with a chronic leg ulcer roughly once a month, no more often than that’. (GP 8)

### Responsibility for diagnosis

In general, the GPs considered venous leg ulcers as being the most common type of wound that they diagnosed, but they also mentioned arterial leg ulcers and diabetic ulcers.

The informants emphasised the importance of diagnosing the wound and that it was considered an important part of the GP’s work to give a medical diagnosis and thus ensure proper treatment for the patient. However, the routines surrounding the diagnosis process were perceived by the GPs as being unclear, and they were not certain if all patients who came to the healthcare centre for wound dressing were actually given a medical diagnosis of their wounds:

‘there was a patient who came here for a year for wound dressing but it wasn’t until he sought an emergency consultation that an infectious diseases doctor realised that the wound had not been assessed and he was then given an assessment and received surgery for venous insufficiency and then the wound healed … so I think we have a huge gap to fill there’. (GP 11)

In addition to the lack of routines for diagnosis, the diagnosis process was also complicated by the fact that the GPs became involved late in the process without having insight into the wound management. One concern highlighted by the GPs was that they were only consulted when there was an unexpected problem in wound treatment, such as a wound infection, and it was therefore pointed out that one reason for patients sometimes not having an aetiologic diagnosis of the wound could be because they had not been further assessed by the GP:

‘the problem we have is that the patients remain with the district nurse and do not come to the doctor for further assessment of the underlying aetiology … my task is to further assess what it is due to and intervene in cases of venous insufficiency or arteriosclerosis and whatever can be done …’. (GP 11)

Lack of clarity in the diagnosis process was highlighted and the informants expressed uncertainty regarding the assessment procedure. Some patients were considered to have received an adequate wound assessment whilst in other cases this was described as arbitrary with regard to routines and collaboration with DNs. The patient being given an adequate assessment of their wound was considered to be dependent on optimal collaboration between the GP and the DN:

‘our diagnosis process is probably a bit wanting … how do we do this exactly? I can in fact say that I don’t really know, I think it is very different from case to case. I think some come to the doctor and receive a clear assessment, a clear diagnosis and a good plan. The collaboration between the GP and the nurse works well, and sometimes they just come in [patients] by chance or drop by or you know and so then it can be a bit hit and miss. There are no good written routines for this’. (GP 7)

Making a diagnosis was generally considered by GPs to be a demanding part of their work, requiring resources and time. The GPs considered Doppler assessment to be an essential resource as it is important to determine the degree of arterial circulation:

‘on the arterial side we usually check the ankle index to see if we can give compression therapy… I think it is difficult … how much compression can be given? we do a basic check of the ankle pressure and then decide what compression to give’. (GP 16)

Time was issue that was raised by the GPs who reported that they were often called during emergency appointments to assess wounds, which meant that they did not have time to study the patient’s medical history in great detail:

‘it is often not that easy as the leg is usually swollen and it can be a bit difficult to assess the arterial pulse and they often come in for an emergency appointment so they are not one of your own patients who you know’. (GP 6)

The informants highlighted the importance of patients being booked in to see the regular GP who knows the medical history of the patient, rather than this being done in passing by the duty doctor.

### Desire for continuity

The desire for continuity concerning a number of aspects was clearly described. The importance of continuity in the contact between DN and patient, and between DN and GP was raised. Several advantages were believed to result from the patient being seen by the same staff. The relationship between the patient and the DN was thought to be particularly important for elderly patients and those who have difficulty in describing their problems, since there could be a number of contextual factors that affect wound healing. Continuity was considered to benefit the overall care and facilitated the monitoring of wound healing. It was generally regarded as difficult if the GP had to assess the patient’s wound solely through reading the DNs records in the medical notes without actually seeing the patient. One of the informants had clinical experience of the importance of continuity for wound healing and described it in the following way:

‘I think it is continuity for the patient, often when there is a change of nurse in connection with wound dressing I notice that it is not as good, that is my personal experience, I think that if the same person does it and there is continuity then its better, everyone does things in a slightly different way, I notice if we have a locum nurse for a few days then it is not as good’. (GP 9)

Continuity also entails aspects of communication. Discussion with a DN before starting an intervention was considered to be crucial for achieving the best possible result. In this context, it was seen as important to have common treatment goals and treatment plans, which would allow continual assessment of wound healing. The informants expressed concern that such discussions did not currently happen. It was not always the same DN and GP who assessed the patients over the course of the treatment. This was seen to be particularly important with regard to chronic leg ulcers which were slow-healing, which according to the informants required input from various healthcare professions in order to view the problem from the perspectives of different professionals who could then contribute with different knowledge and skills. It was thought that it could lead to better assessment, and more thorough and more satisfactory treatment of the patient:

‘if there are several of us it would be better if we collaborated, I mean in general, when I started as a GP then I was lacking in experience … just to discuss patients because when you discuss something then you can get new ideas, there can be a lot of problems and it can resolve a great deal [collaboration] … just saying something out loud then you hear yourself and come up with an idea, so I think it is really important’. (GP 10)

One difficulty raised by the GPs was when they were called to assess a chronic leg ulcer while they were busy seeing other patients booked into their clinics. They perceived their work as fragmented and the assessments were carried out without having the opportunity to get to know the patients better.

### Collaboration within the organisation

The informants emphasised that wound management is an area that is improved through GPs and DNs working together. Collaboration between professions was thought, however, to be largely dependent on organisational factors. The informants believed that the DNs had taken on a lot of the care of these patients independently because of low staffing levels among the GPs. Stability in staffing levels of both GPs and DNs was seen as a basis for collaboration and for both professions to find their positions and roles in wound care:

‘… it is the lack of staff. It’s not that we aren’t willing to collaborate, I really don’t think so, instead we want to do a good job, and we don’t have any problem talking to each other, it’s not that. But it is when it is so fragmented and so urgent’. (GP 5)

The informants raised the issue of staff turnover among DNs. Many of the experienced DNs had left under a short space of time, which affected the provision of healthcare and was also felt to have a huge impact on collaboration. The informants pointed out that it takes time to build a working relationship and good collaboration regarding wound care.

The difficulty with different DNs taking care of patients’ wounds was that they were not so well informed about the patients’ medical history and background. This influenced the assessment and made it impossible for both professions to build an effective collaboration around patients with wounds:

‘difficult because it … it lacks structure here, right now and, what can I say … there aren’t any such routines … at the moment it is not the same person who sees the patient every time … it’s not the same nurse … instead it’s different ones’. (GP 6)

Lack of time was also reported as a difficulty and made it hard to gain a proper insight into the patient’s medical history. This resulted in the GPs quickly inspecting the leg ulcer and making an on the spot assessment, which was thought to have consequences for the aetiologic diagnosis. It was therefore deemed important that an experienced DN took care of the treatment:

‘often it is the duty doctor who has to go and quickly look at the leg ulcer. It is an on the spot assessment, so you need to have reasonably good knowledge and a nurse who is experienced in clinic work’. (GP 13)

The informants pointed out that the GP and DN teams that had previously existed had been withdrawn due to a change in reimbursement models. The result of this was that both professions worked in isolation without any easy way of sharing the work. The GP’s workload, where as many patients as possible are booked in, and the DN’s challenging situation with home care visits was emphasised. The desire for a flatter organisation that could lead to more teamwork and shorter decision-making pathways was obvious:

‘the team that we had previously is now gone … the GP is a lone wolf who should see many patients whilst at the same time our district nurses have to struggle with home care and try to meet the demands … it becomes a sense of us and them and we have no straightforward way to share the work between us as there was before so that I think it is a shame, I hope that the organisation will become flatter and that all patient visits give roughly the same reimbursement and that would lead to more teamwork’. (GP 11)

The informants felt that it was the responsibility of the healthcare centre’s management to develop an efficient organisation where there was a structure for working with patients with wounds. Some informants worked at healthcare centres where wound care groups had been formed, which were thought to facilitate collaboration between the professions. In these groups, healthcare assistants and DNs took care of most of the work with patients with wounds and the GP responsible for the patients was called in when required. The wound care group was also tasked with keeping themselves and other colleagues up to date about developments in wound care.

Having specific GPs connected to the home care service, where many patients with wounds are cared for, was considered to facilitate collaboration between the professions and was thought to lead to greater continuity and better routines regarding wound care, and consequently better care of the patients. The informants emphasised the need for a stable relationship between GP and DN, and considered this important for collaboration and for security of responsibility.

## Discussion

The GPs in this study perceived their role in wound care primarily as that of consultant and coordinator, making their part of the treatment fragmented. The consequence of the GPs acting only as consultants may be that they distance themselves from wound treatment. They reported that patients continued to see the DN without any further assessment of the wound by the GP. Lack of medical assessment and proper diagnosis of the wound may therefore lead to poor practice, with the result that the patient suffers prolonged wound healing. However, GPs underlined that they should engage more in wound treatment since they struggled to make a diagnosis and therefore give appropriate treatment. Striving for continuity in treatment was raised and was supported by the fact that GPs had noted increased compliance with treatment if the same DN took care of the wounds. This was considered to benefit the patient as it resulted in more effective and faster wound healing. GPs perceived their work situation as being time-pressured and there was a lack of organisational structure with unclear areas of responsibility concerning wound care. Clarity regarding responsibilities in wound care could affect the GPs’ ability to diagnose the wounds and it is important that this is considered at the organisational level. Unstructured organisation can also affect patient safety due to GPs having to make hasty assessments, which further emphasises the organisation’s responsibility. Collaboration with the DNs was, to a large extent, influenced by organisational factors, linked in particular to the instability of staffing levels and lack of time. The results therefore indicate the need for organisational support to enable collaboration between the professions.

## Discussion of results and comparison with existing literature

The results show that the patient’s regular contact was with the DN responsible for wound treatment and that the GPs regarded their role in wound care as that of a consultant. Earlier research has shown that GPs rely on the expertise of DNs in wound treatment, but less is known of how GPs regard their own role in wound care (eg, Sadler *et al*., 2006). The role of consultant means, according to the GPs, in this study, that in most cases they are called in if there is an unexpected problem, for example, if the wound is infected and there is a need for antibiotics.

The underlying aetiologic diagnosis is the basis of wound care and for establishing a treatment plan for the patient and thus avoid giving the wrong treatment (SIGN, 2010; Mooij and Huisman, 2016; VISS, 2016). Since DNs are considered to have a leading role in wound care, this places great demands on them and they need an increased awareness of the importance of an aetiologic diagnosis as a basis for the wound care, in particular since earlier research has shown that many wounds are treated without a diagnosis (Friman *et al*., 2010; Mooij and Huisman, 2016). It was clear that the GPs regarded making a diagnosis of the wound as important, but the lack of clear routines and guidelines concerning the diagnosis process meant that this was not always carried out. A lack of routines and guidelines for wound diagnosis can lead to a lack of clarity regarding responsibility in wound care (Mooij and Huisman, 2016; Guest *et al*., 2017). A number of factors made the diagnosis process more difficult. One reason cited was that the patients initially asked to see the DN. Another reason given by the informants was that the patients were referred to the DN by the healthcare centre staff. The GPs believed that the patients then continued seeing the DNs without involving GPs. The results indicate that there is a need for a change in routines and procedures concerning wound care where the GPs need to be involved earlier in the patient’s treatment. It has been shown and discussed in recent studies that structured care for patients with leg ulcers, with clear professional roles and responsibilities, leads to more effective and faster wound healing (Öien *et al*., 2016; Guest *et al*., 2017). The reduction in healing times can therefore be explained by the provision of structured care, including assessment and diagnosis, which is a pre-requisite for optimum care and continuity in treatment until the wound heals.

The consulting role of GPs was not always advantageous and a desire for continuity was raised. The DNs should therefore engage the GPs earlier into the wound care process and strive for continual dialogue between the professions. In this study, it is clear that continuity between both patient and DN and between the professions was regarded as important. It was seen as particularly important with regard to elderly patients who cannot describe their problems. This has also been highlighted by the national medical assessment agency (SBU, 2014). The DN’s close contact with the patient, and the continuity of treatment were regarded by the GPs as contributing to a more holistic view of the patient. It was also noted by the GPs that wound healing can be improved if the same DN changes the dressing each time. This has also been demonstrated in a previous study, highlighting the importance of the same DN meeting the patients and regularly evaluating the treatment (Friman *et al*., 2011). This is in line with Edwards *et al*. (2013) who further noted that providing continuity and standardisation of care was the best way to obtain optimal outcomes for adults with leg ulcers.

A large part of the collaboration between professions was thought to be dependent on organisational factors. The lack of stability in staffing levels was reported as a very influential factor, which undermined the fundamental possibilities for collaboration between the professions. Stable staffing levels were considered vital for professions to be able to find their respective roles in collaboration and should also contribute to clarity in the division of responsibilities as is advocated in the literature (Jansen, 2008; Xyrichis and Lowton, 2008; Moore *et al*., 2014; McInnes *et al*., 2015). Time pressure is another organisational factor that was clear from the results. This emerged when the informants described how they assessed wounds when they were duty doctor. These assessments were made more difficult by the fact that different DNs took care of the patient’s treatment and this meant that there was also a lack of continuity in the care. The tasks of primary care are politically determined in Sweden (Swedish National Board of Health and Welfare [Socialstyrelsen], 2011), which was pointed out by some informants. They discussed political decisions that had led to teamwork being withdrawn, with the result that there was no longer any natural collaboration between GP and DN. Progress with organisational planning, as is recommended in the literature (Jansen, 2008; Xyrichis and Lowton, 2008), was described when specific GPs were linked to home care. This resulted in better collaboration between the professions and continuity of treatment of the patient.

## Study limitations

Taken together, the variety in the sample of informants provides a broad picture of GP perspectives. The interviews were carried out using an interview guide, which had been tested in five pilot interviews; these interviews were carried out by two registered nurses with experience of primary care. Their experience from primary care meant that they had both knowledge and an understanding of the research area, which was considered an advantage. The pilot interviews resulted in small adjustments to the interview guide. The researchers represent different perspectives: nursing, medicine and education, allowing a broader understanding of the phenomena and the possibility to capture the nuances of statements. The first author is a DN with considerable experience of wound management. This can be seen as an advantage in being able to obtain nuanced data material. However, there is also a risk that the data analysis is influenced by personal interpretations, making it necessary to maintain a professional distance to the perspectives of the informants (Kvale and Brinkmann, 2009). One of the authors belongs to the same professional group as the informants. This was considered to contribute to richer data material since a common professional culture and the trust of colleagues can influence the relationship between the informants and the interviewer (Coar and Sim, 2006). Since this author works as a dermatologist at a university hospital, the interview situation may contribute to asymmetry in the power relations (Kvale and Brinkmann, 2009). The challenge for the interviewer here is to withhold any preconceptions and allow the informants to present their own experiences. The risk otherwise is that the interviewers feel they are being questioned and that the interviewer is guided by their own opinions (Chew-Graham *et al*., 2002).

## Conclusions

The GPs perceive their role in wound care as that of a consultant in a domain in which the DNs set the agenda. They feel that they become involved far too late in the patient’s treatment, which may have consequences for the underlying aetiologic diagnosis and thereby prolong healing time. There were shortcomings concerning collaboration, which to a large extent were attributed to organisational factors. A change in working methods where GPs are involved earlier in wound care would probably encourage collaboration between professions and lead to faster healing of leg ulcers and thus benefit the patient care.

## Further research

This present study indicates the need for studies on how to improve organisational structures with clear professional roles and responsibilities in wound treatment. Therefore, it is important to further study how to improve GPs role in wound care. A further area of interest is to study collaborative structures between GPs and DNs in wound care as this is supposed to improve wound healing.
